# High-resolution axial MR imaging of tibial stress injuries

**DOI:** 10.1186/1758-2555-4-16

**Published:** 2012-05-10

**Authors:** Takeo Mammoto, Atsushi Hirano, Yohei Tomaru, Mamoru Kono, Yuta Tsukagoshi, Sinzo Onishi, Naotaka Mamizuka

**Affiliations:** 1 Department of Orthopaedic Surgery and Sports Medicine, Tsukuba University Hospital Mito Medical Center, Mito Kyodo General Hospital, 3-2-7, Miya-machi, Mito, Ibaraki, 310-0015, Japan

**Keywords:** Tibial stress injury, High resolution MR imaging, Stress fracture, Shin splint

## Abstract

**Purpose:**

To evaluate the relative involvement of tibial stress injuries using high-resolution axial MR imaging and the correlation with MR and radiographic images.

**Methods:**

A total of 33 patients with exercise-induced tibial pain were evaluated. All patients underwent radiograph and high-resolution axial MR imaging. Radiographs were taken at initial presentation and 4 weeks later. High-resolution MR axial images were obtained using a microscopy surface coil with 60 × 60 mm field of view on a 1.5T MR unit. All images were evaluated for abnormal signals of the periosteum, cortex and bone marrow.

**Results:**

Nineteen patients showed no periosteal reaction at initial and follow-up radiographs. MR imaging showed abnormal signals in the periosteal tissue and partially abnormal signals in the bone marrow. In 7 patients, periosteal reaction was not seen at initial radiograph, but was detected at follow-up radiograph. MR imaging showed abnormal signals in the periosteal tissue and entire bone marrow. Abnormal signals in the cortex were found in 6 patients. The remaining 7 showed periosteal reactions at initial radiograph. MR imaging showed abnormal signals in the periosteal tissue in 6 patients. Abnormal signals were seen in the partial and entire bone marrow in 4 and 3 patients, respectively.

**Conclusions:**

Bone marrow abnormalities in high-resolution axial MR imaging were related to periosteal reactions at follow-up radiograph. Bone marrow abnormalities might predict later periosteal reactions, suggesting shin splints or stress fractures. High-resolution axial MR imaging is useful in early discrimination of tibial stress injuries.

## Background

Stress-related injuries occur frequently during sports activities, and most stress injuries involve the tibia [1]. These injuries comprise a wide spectrum of bone abnormalities that occur in response to chronic repetitive stress applied to normal bone [2]. Early discrimination of tibial stress injuries is crucial to allow athletes to return sports activities.

Magnetic resonance (MR) imaging has emerged as a highly sensitive method for detecting bone stress injuries [3-7]. MR imaging allows depiction of abnormalities before the development of radiographic or CT abnormalities [6-8]. Sensitivity and specificity in MR imaging are superior to nuclear scintigraphy for detection of osseous abnormalities [6]. Therefore MR imaging is considered the gold standard for the diagnosis of stress injuries [3,5,6].

MR imaging also provides a detailed anatomic evaluation of the regional tissues including the periosteal tissue, endosteal tissue, and cortical bone. MR imaging of tibial stress injuries shows periosteal edema, bone marrow edema, and fracture lines [6,9-11]. MR imaging using a microscopy coil provides high-resolution MR images of extremities [12]. High-resolution MR images with a microscopy coil are superior to those with conventional coils in terms of spatial resolution, signal-to-noise ratios, and contrast-to-noise ratios [12].

The purpose of this study was to evaluate the relative involvement of tibial stress injuries using high-resolution axial MR imaging, and the correlation between MR imaging and radiographic changes.

## Methods

Between January 2008 and November 2009, 33 patients (14 males, 19 females; mean age 16 years) were enrolled in this study. They suffered lower leg pain in the middle or distal portion of the medial aspect of the leg during or after sports activities, and with moderate or severe tenderness along the medial posterior border of the tibia.

All patients were evaluated with radiographs at the time of initial examination and approximately 4 weeks later, using anterior-posterior and lateral radiographs of the leg. Patients were divided into 3 groups according to radiographic abnormally: Group 1, no radiographic abnormalities on both initial and follow-up examination; Group 2, no radiographic abnormalities at initial examination, but local periosteal reaction or callus formation at follow-up; and Group 3, periosteal reaction at both initial and follow-up examination. Informed consent was obtained from the patient for publication of this research and any accompanying images.

### High-resolution axial MR imaging

All MR imaging was done on a 1.5T unit system (Magnetom Symphony, Siemens, Germany) with a 23-mm microscopy surface coil (Loop Flex coil, Siemens, Germany). The microscopy surface coil was positioned over the site of maximal tenderness of the leg examined.

All MRI examinations included the following: axial turbo spin echo (TSE) proton density-weighted images (PD) (repetition time (TR) ms/echo time (TE) ms = 3000/16), axial gradient recalled echo (GRE) T2*-weighted images (T2*) (TR/TE/flip angle = 500/25/30), and axial TSE fat-suppressed T2-weighted images (T2FS) (TR/TE = 4000/121). The field of view (FOV) was 60 × 60 mm, and the slice thickness was 2.0 mm with intersection gap, an imaging matrix was 256 × 256.

The image evaluation included abnormal signals of the periosteum, bone marrow, and cortical bone. Edema along fascial structures and involving muscle were noted. Images were not blindly examined by two orthopaedic surgeons (TM and AH). The location of abnormalities in the axial plane was defined as anterior, posterior, medial, and lateral, dividing the tibial cortex into four parts using two orthogonal crossing lines [6]. The area of bone marrow with signal abnormalities in the axial MR imaging was defined as partial or complete bone marrow abnormalities. Differences between groups at duration from symptom to examination were assessed using ANOVA with Tukey post hoc test. For the rate of MRI abnormality, statistical analysis was performed using Chi square tests. Statistical significance was set at *p* < 0.05 for all comparisons.

## Results

Patients suffered lower leg pain a mean of 5 weeks (range, 1 to 12 weeks) before initial radiographic examination. The mean time between the initial radiograph and MR imaging was 29 days (range, 2 to 62 days).

Twenty-six of 33 patients showed no periosteal reaction, callus formation, or fracture line at initial examination. Nineteen of 26 that did not show radiographic abnormalities at follow-up were assigned to Group 1. Seven patients that showed local periosteal reaction or callus formation at follow-up were assigned to Group 2, and 7 patients that showed periosteal reaction at the initial examination were assigned to Group 3 (Table 1).

**Table 1:** Characteristics of each group profile

	Number of patients	Time between symptom onset and initial X-ray (weeks)	Time between initial X-ray and MRI examination (days)
G1	19	4.8 (1–10)	17.3 (2–62)
G2	7	4.9 (1–12)	25.8 (8–50)
G3	7	8.4 (1–12)	17.3 (5–35)

G1, G2 and G3 indicate Group 1, 2 and 3. No significant differences between groups in duration from symptom to radiographic examination, and from radiographic to MRI examination.

**Figure 1:**
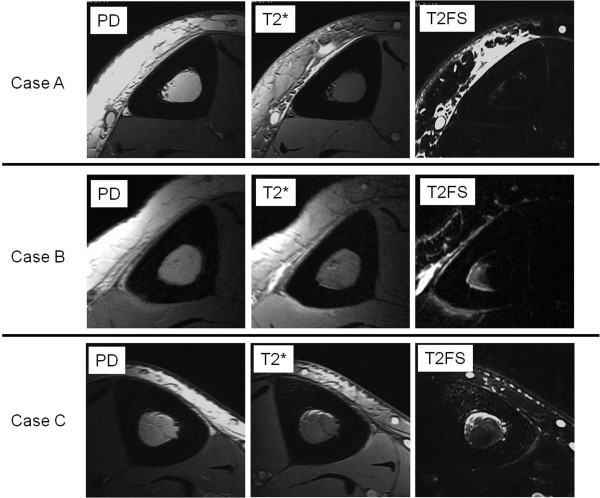
**High-resolution axial MR imaging of Group 1.** Top: Case A showing abnormal periosteal signals along the medial surface of the tibia. High-resolution MR imaging shows abnormal signals along the anterior-medial aspect of the tibial bone marrow. Middle: Case B showing abnormal periosteal signals along the medial and medial posterior surface of the tibia. MR imaging shows abnormal signals in medial-posterior part of the tibial bone marrow, but never extends to the entire bone marrow. Bottom: Case C showing abnormal periosteal signals along the posterior surface of the tibia. MR imaging shows abnormal signals in anterior-medial part of the tibial bone marrow.

For 19 patients in Group 1, the mean period of symptoms was 4.8 weeks (range, 1 to 10 weeks). MR imaging was taken a mean of 17.3 days (range, 2 to 62 days) after initial radiographic examination. In 18 of 19 patients, the axial high-resolution MR imaging showed abnormal periosteal signals along the medial or medial-posterior surface of the tibia. Seventeen of 19 legs showed abnormal signals along the anterior-medial aspect of the tibial bone marrow; these abnormal signals were noted remarkably on fat-suppressed T2-weighted images. This high signal area represented less than half of the bone marrow in 11 patients and exceeded half of the bone marrow in 8 patients. However this abnormal area never extended throughout the entire bone marrow (Figure 1).

**Figure 2:**
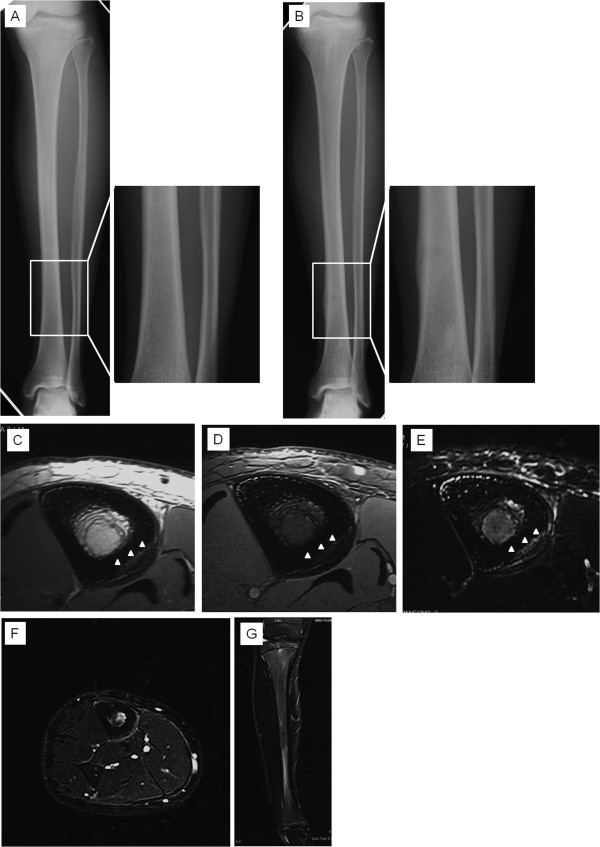
**Radiographic and MR imaging of Group 2.** [A] Initial radiographic examination shows no periosteal reaction. [B] Follow-up radiographic examination shows local peroneal reaction. High-resolution axial MR imaging using proton-density WI [C], T2* WI [D] and fat-suppressed T2 WI [E] shows abnormal periosteal signals along the medial-posterior surface of the tibia. Abnormal bone marrow signals exceed the entire tibial bone marrow. MR imaging shows cortical abnormal signals in the medial-posterior part of the tibia (arrow heads). Axial [F] and coronal [G] fat-suppressed T2 WI MR imaging using conventional coil shows whole tibial bone marrow and surrounding soft tissues. High-resolution imaging is useful to detect signal changes of the regional tissues.

The 7 patients in Group 2 experienced lower leg pain for a mean of 4.9 weeks (range, 1 to 12 weeks). MR imaging was done at a mean of 25.8 days (range, 8 to 50 days). All patients showed abnormal periosteal signals along the medial or medial-posterior surface of the tibia. On fat-suppressed T2-weighted images, abnormally high bone marrow signals exceeded the entire tibial bone marrow in all patients. In the cortex, 3 patients showed abnormal signals in the anterior part and 6 showed abnormal signals in the medial-posterior part of the tibia (Figure 2).

**Figure 3:**
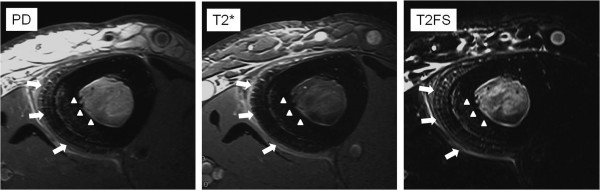
**High-resolution axial MR imaging of Group 3.** MR imaging shows abnormal periosteal signals along the medial and medial-posterior surface of the tibia. Abnormal bone marrow signals extend through the entire bone marrow. Abnormal cortical signals are observed in the medial-posterior cortex (arrow heads). Periosteal hypertrophy is observed in the medial and medial-posterior aspect (arrow), indicating radiographic periosteal reaction.

The remaining 7 patients in Group 3 experienced pain for a mean of 8.4 weeks (range, 1 to 12 weeks), and MR imaging was taken at a mean of 17.3 days (range, 5 to 35 days) after initial examination. Abnormal signals in the periosteum were seen in 6 patients. The entire bone marrow was seen as abnormal in 3 patients, and all showed patients showed abnormalities in the medial-posterior cortex. Four patients showed partial abnormal signals in bone marrow and 2 showed abnormal signals in the medial-posterior cortex. Abnormalities in the anterior cortex were seen in 3 patients (Figure 3).

Statistical comparison of the rate of abnormalities among Groups 1, 2 and 3 showed significant differences in abnormally bone marrow signals (*p* < 0.05 in partial bone marrow abnormality (Group 1: 89.5% vs Group 2: 0%) and *p* < 0.05 in entire bone marrow abnormality (Group 1: 0% vs Group 2: 100%), and medial-posterior cortical bone signals (*p* < 0.05 (Group 1: 10.5% vs Group 2: 85.7%)) (Table 2).

**Table 2:** Characteristics of MRI abnormality in each group

	Abnormally periosteal signals	Abnormally partial bone marrow signals	Abnormally entire bone marrow signals	Abnormally signals in anterior cortex	Abnormally signals in medial-posterior cortex
G1	18/19	17/19	0/19	2/19	2/19
	(94.7%)	(89.5%) ^*a*^	(0%) ^*b*^	(11%)	(10.5%) ^*c*^
G2	7/7	0/7	7/7	3/7	6/7
	(100%)	(0%) ^*a*^	(100%) ^*b*^	(42.9%)	(85.7%) ^*c*^
G3	6/7	4/7	3/7	3/7	5/7
	(85.7%)	(57.1%)	(42.9%)	(42.9%)	(71.4%)

MRI abnormalities including periosteal signals, bone marrow signals and cortical bone signals are shown. Significant differences are found in bone marrow signals (*a* and *b*), and medial-posterior cortical bone signals (*c*) (*p* < 0.05).

## Discussion

In the current study, partially localized abnormal signals in the bone marrow on axial MR imaging was found even among patients with no abnormalities at both initial and follow-up radiographic exams. Abnormal signals extending throughout the entire bone marrow consistently showed radiographic abnormalities in later images. MR imaging with the initial radiographic abnormalities showed partial or entire bone marrow abnormalities. Thus, the area of abnormal signals in the tibial bone marrow in the axial MR imaging might predict later radiographic changes. In addition, abnormalities in the medial-posterior cortex in the axial MR imaging tended to be related to radiographic changes.

Group 1 showed negative finding on examination in both initial and follow-up radiographs, which recognized as shin splint. Group 2 showed positive findings in follow-up radiograph that recognized as stress fracture. Positive finding in initial radiograph in Group 3 was not clear which results of stress fracture or acute injuries on previous stress fracture site. This study showed the characteristic axial MR imaging of tibial stress injuries in each condition. There were no differences in MR images according to middle or distal part of tibia.

Aoki reported that coronal MRI scans can distinguish stress fractures and shin splints before radiographs show a detectable periosteal reaction of the tibia [9]. Abnormally high signals in the bone marrow on short tau inversion recovery (STIR) or fat-suppressed MRI are wide and continue to the cortex in stress fractures; however abnormally high linear signals in the bone marrow are seen in shin splints. Ahovuo reported that detection of stress injuries in axial images of the tibial shaft shows higher rates of diagnostic accuracy than in coronal images [13]. This study showed that an axial MR imaging detected relative involvement in the periosteum, bone marrow, and cortical bone.

In the current study, abnormal periosteal signals were found in the medial or medial-posterior surface of the tibia in all groups. This observation might be related to traction periostitis along the insertion of the soleus fascia, flexor digitorum longus, and tibialis posterior, implying a potential cause of shin splints [14-16].

Partial bone marrow abnormalities did not involve periosteal reactions at follow-up radiographs in this study. Similarly, abnormal high linear signals on STIR in coronal MRI scans are related to shin splints [9]. This abnormally high signal intensity in bone marrow might be secondary to edema or hemorrhage related to microdamage and the associated repetitive response. Johnell reported that the metabolic activity in the biopsy bone was increased at the medial edge of the tibia with shin splints [17]. Shweitzer reported that an increase in bone marrow signal intensity on MR imaging was caused by altered weight-bearing [18]. This partial bone marrow abnormality might be related to changes in load and metabolism of tibial bone marrow.

Abnormalities in the entire bone marrow were related to abnormalities in the medial-posterior cortex of the tibia on axial MR imaging. Stress fractures show intramedullary abnormal signal continuing with the cortex of the tibia on coronal MRI scan [9]. This cortical abnormality might predict a periosteal reaction such as a stress fracture, as well as the area of abnormal signals in bone marrow.

The relationship between stress fracture and shin splint is controversial. Aoki et al. reported that patients with shin splints did not have a stress fracture at the late phase. Therefore they think that shin splints may have some relation to stress reaction of bone but are likely different clinical entities from stress fractures. Other authors reported that shin splints are part of continuum of fatigue damage of stress fracture [2,5].

Anderson et al. reported that stress fractures shows abnormal marrow signal intensity with linear signals abnormally extending through the anterior cortex with periosteal fluid [2]. Fredericson et al. reported that periosteal edema reflects periostitis or shin splints [5]. Abnormal linear signals involving the cortical surface are thought to represent a relatively early physiologic response of bone to stress [5]. More severe edema of both the periosteum and bone marrow are found to represent increasing severity with periosteal edema [5]. Therefore they concluded that periosteal edema may be seen as the initial injury on the spectrum, which, if allowed to progress, would evolve into a more serious bone injury [5].

In the current study, no patients with periosteal abnormalities and partial bone marrow abnormalities on axial MR imaging showed findings of periosteal reaction on follow-up radiograph. Patients with abnormalities in the entire bone marrow or medial-posterior cortex showed periosteal reaction on follow-up radiograph. This finding suggests that periosteal reaction on follow-up might represent the initial response of the tibia to repetitive stress. The bone marrow abnormality initially occurs in part of the bone marrow, and then extends to the entire tibia if the abnormally relates to micro damage of the bone marrow. The medial-posterior cortex might react to the stress and a periosteal reaction occurs if the damage to the bone marrow expands to the entire bone marrow.

Conventional radiographs are the primary tool for diagnosing suspected stress injuries; however, radiographic findings sometimes lag behind the MR imaging [7]. The MR findings with initial radiographic abnormalities might represent a subsequent reaction following initial findings. This subsequent reaction might represent the healing course of edema or hemorrhage related to microdamage.

There are some limitations to this study. The first limitation is that the interval between the symptom onset and MR imaging was not definite. However the mean duration of symptoms before MR imaging was not different in Groups 1 and 2. In addition, the duration between radiographs and MRI did not show differences. Therefore we believe that findings in MR imaging are available for early discrimination of tibial stress injuries. The second limitation is that follow-up MR imaging was not evaluated in this study. The MR findings with initial radiographic reaction were thought to represent a reaction to tibial stress injuries. However a study including consecutive MR imaging is needed in the future. Despite these limitations, there have been no reported studies of tibial stress injuries using high resolution MR imaging.

## Conclusions

This study showed that bone marrow abnormalities on high-resolution axial MR imaging were related to periosteal reactions on follow-up radiographs. Partial abnormal signals in bone marrow were not related to follow-up periosteal reactions and entire bone marrow abnormalities showed periosteal reactions later. This finding suggests that bone marrow abnormalities might predict later periosteal reactions indicating shin splints or stress fractures. High-resolution MR imaging is useful in the early discrimination of tibial stress injuries.

## Competing interests

The authors declare that they have no competing interests.

## Authors’ contributions

TM conceived of the study, and participated in its study and coordination. AH was one of the leading investigators, participated in the sequence alignment and drafted the manuscript. YT, MK, YT and SO were involved as well in the study practically. NM participated in the sequence alignment. All authors read and approved the final manuscript.
